# Modulation of the Kynurenine Pathway in Obese Mexican Navy Women Following a Structured Weight Loss Program: A Pre–Post-Intervention Study

**DOI:** 10.3390/nu18020211

**Published:** 2026-01-09

**Authors:** Laura Sánchez-Chapul, Daniela Ramírez-Ortega, María Alejandra Samudio-Cruz, Elizabeth Cabrera-Ruiz, Alexandra Luna-Angulo, Gonzalo Pérez de la Cruz, Jesús F. Valencia-León, Paul Carillo-Mora, Carlos Landa-Solís, Edgar Rangel-López, Abril Morraz-Varela, Marco Tulio Romero-Sánchez, Verónica Pérez de la Cruz

**Affiliations:** 1 Neuromuscular Diseases Laboratory, Clinical Neurosciences Division, National Institute of Rehabilitation “Luis Guillermo Ibarra Ibarra”, Mexico City 14389, Mexico; lunangulo@gmail.com (A.L.-A.); avril.morraz@gmail.com (A.M.-V.); 2 Neurobiochemistry and Behavior Laboratory, National Institute of Neurology and Neurosurgery “Manuel Velasco Suárez”, Mexico City 14269, Mexico; danielaramirez@innn.edu.mx; 3 Clinical Neurosciences Division, National Institute of Rehabilitation “Luis Guillermo Ibarra Ibarra”, Mexico City 14389, Mexico; psic.alejandra.samudio@gmail.com (M.A.S.-C.); neuropolaco@yahoo.com.mx (P.C.-M.); 4 Basic Neurosciences Division, National Institute of Rehabilitation “Luis Guillermo Ibarra Ibarra”, Mexico City 14389, Mexico; elicabreraruiz@gmail.com; 5 Department of Mathematics, Faculty of Sciences, Universidad Nacional Autónoma de México (UNAM), Mexico City 04510, Mexico; gonzalo.perez@ciencias.unam.mx; 6 Dirección General Adjunta de Sanidad Naval, Secretaría de Marina Armada de México, Mexico City 04830, Mexico; jesus_ferval@hotmail.com; 7 Tissue Engineering, Cell Therapy and Regenerative Medicine Unit, National Institute of Rehabilitation “Luis Guillermo Ibarra Ibarra”, Mexico City 14389, Mexico; cls_73@hotmail.com; 8 Cell Reprogramming Laboratory, National Institute of Neurology and Neurosurgery “Manuel Velasco Suárez”, Mexico City 14269, Mexico; raledg@hotmail.com; 9 Center for Research in Applied Biotechnology, National Polytechnic Institute, Santa Inés Tecuexcomac 90700, Mexico; mromeros1900@alumno.ipn.mx

**Keywords:** kynurenine pathway, kynurenic acid, 3-hydroxykynurenine, tryptophan, obesity

## Abstract

**Background:** Obesity is characterized by chronic low-grade inflammation and metabolic disturbances, including an altered tryptophan (Trp) catabolism through the kynurenine pathway (KP). Since the KP is closely linked to immune activity, energy metabolism, and hepatic function, modulating its flux through lifestyle interventions has gained interest as a potential therapeutic strategy. **Objective:** This exploratory study aimed to investigate the impact of a structured 12-week weight loss program (WLP) on serum KP metabolites in a sample of Mexican women with obesity. **Methods**: This study involved a pre–post-intervention design conducted in twenty-four women with clinically diagnosed obesity from the Mexican Navy who underwent a structured 12-week weight loss program combining a hypocaloric diet with moderate-intensity aerobic exercise; no control group was included. Anthropometric parameters, serum biochemistry, and circulating levels of Trp, kynurenine (KYN), kynurenic acid (KYNA), and 3-hydroxykynurenine (3-HK) were assessed before and after intervention. Psychological assessments of anxiety and depression were also conducted in a subset of participants. **Results**: The WLP significantly reduced body weight, BMI, fat mass, fasting insulin, and C-reactive protein levels. Serum concentrations of Trp, KYN, and KYNA decreased, while 3-HK showed a non-significant upward trend. Enzymatic indexes revealed a significant increase in the 3-HK/KYN ratio and a decrease in the KYNA/3-HK ratio, suggesting a shift toward kynurenine monooxygenase (KMO) branch. Notably, higher KYNA-related ratios were inversely associated with depressive symptoms. **Conclusions**: These findings position the KP as a responsive metabolic interface potentially linking improvements in body composition, liver function, and psychological status during structured weight loss efforts.

## 1. Introduction

Obesity is a major global health concern that has reached epidemic proportions [1]. According to the World Health Organization (WHO), in 2022, more than one in eight people globally (approximately 890 million adults) were classified obese [1]. In Mexico, the situation is particularly severe; the 2022 Mexican National Health and Nutrition Survey reported an obesity prevalence of 41.0% among women and 32.3% among men [2,3]. While obesity is caused by an imbalance between calorie intake and energy expenditure, emerging evidence indicates that overeating or physical inactivity alone do not fully explain the complexity of the obesity pandemic [1]. Obesity is now recognized as a chronic, multifactorial disease involving complex interactions among an obesogenic environment and psychosocial, genetic, metabolic, and immunological factors. A hallmark of obesity is chronic low-grade inflammation (cLGI) which affects white adipose tissue (WAT), skeletal muscle, pancreatic islets, and liver [4]. This localized inflammation can escalate into systemic inflammation, contributing to insulin resistance, metabolic dysfunction, and alterations in tryptophan (Trp) catabolism [5].

Trp is an essential amino acid obtained exclusively through the diet. Therefore, its plasma concentration and its brain availability depend on dietary intake, as well as physiological factors such as stress, physical activity, and immune system activation [6]. About 90–95% of circulating Trp is catabolized via the kynurenine pathway (KP), which plays a central role in immune regulation and energy metabolism. The hepatic enzyme tryptophan 2,3-dioxygenase (TDO) initiates this process by catalyzing the rate-limiting step, converting Trp to kynurenine (KYN) [7]. This pathway also produces other metabolites linked to neurodegenerative and metabolic complications associated with obesity [8]. KYN is further metabolized into several downstream metabolites, including kynurenic acid (KYNA), 3-hydroxykynurenine (3-HK), xanthurenic acid (XA), picolinic acid (PA), anthranilic acid (AA), quinolinic acid (QUIN), and ultimately nicotinamide adenine dinucleotide (NAD^+^), an important coenzyme involved in cellular energy homeostasis [9].

In peripheral tissues, the KP can also be activated by the indoleamine 2,3-dioxygenase (IDO1 and IDO2) enzymes, which are upregulated by inflammatory mediators such as interferon-gamma (IFN-γ), tumor necrosis factor alpha (TNF-α), and lipopolysaccharides (LPS), [10,11,12,13]. In the context of obesity, chronic inflammation stimulates IDO1 expression in adipose tissue, resulting in increased KYN levels and an elevated KYN/Trp ratio, both considered markers of inflammation and immune activation [14,15]. As more Trp is diverted toward the KP, serotonin synthesis may be reduced (because tryptophan is the precursor to serotonin), further exacerbating metabolic dysfunction, contributing to appetite dysregulation and possibly favoring to the development of depression [16,17,18,19,20].

Among KP metabolites, KYN activates the aryl hydrocarbon receptor (AhR), a ligand-activated transcription factor involved in immune regulation and xenobiotic metabolism. AhR activation by KYN has been associated with the transcription of inflammatory mediators such as signal transducer and activator of transcription 3 (STAT3) and interleukin-6 (IL-6), both of which are implicated in the development of insulin resistance [19]. In contrast, KYNA demonstrates anti-inflammatory and metabolic regulatory effects. KYNA has been shown to activate G-protein-coupled receptor 35 (GPR35), which promotes thermogenesis and enhances energy metabolism and reduces inflammation in adipose tissue [15,17].

Exercise is a potent modulator of Trp catabolism. Aerobic training increases the expression of kynurenine aminotransferases (KATs) in skeletal muscle, facilitating the conversion of KYN to KYNA and limiting KYN’s ability to cross the blood–brain barrier. This shift not only protects against neuroinflammation but also improves systemic energy balance. Additionally, human studies have demonstrated that caloric restriction and physical activity reduce circulating KYN levels and lower the KYN/Trp ratio [21,22]. Notably, in women with obesity and type 2 diabetes, altered KP activity is characterized by elevated plasma KYNA concentrations and higher KYNA/3-HK ratios [23].

Given this context, we hypothesized that a structured weight loss program integrating a hypocaloric diet, and moderate-intensity exercise would modulate the KP in parallel with improvements in anthropometric, biochemical and behavioral markers. In particular, we anticipated that changes in circulating KYNA levels might reflect a shift toward a more balanced metabolic and immunological state. Modulation of the KP through non-pharmacological strategies such as diet and exercise has attracted increasing interest as a potential approach for restoring metabolic homeostasis. Therefore, the present study aims to investigate changes in serum KP levels of key KP metabolites, including Trp, KYN, KYNA, and 3-HK, in Mexican naval women with obesity participating in a structured weight loss program (WLP) that integrates a hypocaloric diet based on traditional Mexican food and a moderate-intensity exercise regimen.

## 2. Materials and Methods

### 2.1. Participants

Thirty-two women with clinically diagnosed obesity from the Mexican Navy participated in the weight loss program (WLP). Inclusion criteria were an age between 18 and 50 years and a body mass index (BMI) greater than 30 kg/m^2^. Exclusion criteria included psychomotor and medical disorders that could interfere with participation in physical activity (cardiovascular, pulmonary of musculoskeletal disorders). A naval physician conducted a physical and clinical examination before enrollment. None of the participants were engaged in regular or intense training program before entering the WLP. Serum biomarkers and body composition were assessed before and after the 12-week intervention. The presence and severity of depressive and anxiety symptoms were assessed using the Beck Depression Inventory (BDI) and the Beck Anxiety Inventory (BAI), with cut-off points of 10 for the BDI and 6 for the BAI. These instruments are 21-item self-report questionnaires that evaluate characteristic attitudes and symptoms associated with depression and anxiety. Higher scores indicate greater symptom severity. A psychologist from the Mexican Navy administered both inventories individually in a private cabin before and after the WLP.

This study was approved by the Research and Ethics Committees of the Instituto Nacional de Rehabilitación “Luis Guillermo Ibarra” (CONBIOETICA-09-CEI-03120171207). All participants provided written informed consent after being informed of the study’s potential risks and benefits.

### 2.2. Anthropometric Measurements

Body weight, height, and body composition including fat mass, muscle mass, and free-fat mass, were determined using a multi-frequency bioimpedance analyzer (InBody 270, InBody Co., Seoul, Republic of Korea). Participants wore light clothing (shorts) and were instructed to refrain from eating or engaging in physical activity for 3–4 h prior to the assessment. BMI was calculated as weight (kg) divided by height squared (m^2^), with obesity defined as a BMI ≥ 30 kg/m^2^. All assessments were performed in a single session on the same day by a certificated nutritionist from the Mexican Navy, accredited at level 2 by the International Society for the Advancement of Kinanthropometry (ISAK) to minimize measurement variability.

### 2.3. Laboratory Determinations

Blood samples were collected via venipuncture after 12 h over fast using Vacutainer tubes (Beckton, Dickinson and Company, Franklin Lakes, NJ, USA). Serum was immediately separated by centrifugation, aliquoted, and stored at −80 °C until biochemical analysis. Routine Serum biochemistry and liver function parameters were determined at the Clinical Pathology Laboratory of the Naval Medical Center.

### 2.4. Weight Loss Program

The WLP integrated a hypocaloric diet featuring traditional Mexican food with a moderate-intensity physical activity regimen. The dietary and physical activity interventions were designed by a nutritionist and a physician, both from the Mexican Navy. It is essential to note that all participants resided at a single location for the entire 3-month program, ensuring full control over environmental variables. The dietary intervention was personalized, based on each participant’s habitual intake, including the most frequently consumed foods across 3–4 days per week, and was assessed for macronutrient distribution. A calorie-restricted diet was prescribed, comprising 50% carbohydrates, 30% fat, and 20% protein, with a total daily energy intake of 1300 and 1400 kcal, adjusted according to individual requirements. Participants with adherence below 80% were excluded from final analyses. The exercise component consisted primarily of aerobic activity of moderate intensity (3.0–5.9 METs), performed daily [24]. This corresponded to approximate 50–60% of each participant’s heart rate reserve, calculated from baseline assessments. Exercise prescriptions were individualized according to the initial clinical profiles, considering comorbidities, age, BMI, and results from functional evaluations (including electrocardiogram and spirometry), in accordance with established clinical guidelines for adult exercise prescription [25].

### 2.5. Kynurenine Metabolites Determination

KP metabolites were quantified following the protocol described by Wu et al., [26], with minor modifications. Serum samples were deproteinized by mixing with 6% perchloric acid (HCl4) in a 1:1 volume ratio, then centrifuged at 14,000× *g* for 10 min. The resulting supernatants were analyzed via high-performance liquid chromatography (HPLC).

Each KP metabolite was quantified using its corresponding commercially available analytical standard. For each compound, a standard calibration curve was generated. Retentions times for each analyte were confirmed by comparison with their respective standards under identical chromatographic conditions.

Detection of KYN and KYNA was performed using a fluoresce detector (RF-20Axs, Shimadzu, Tokyo, Japan). Excitation/emission wavelengths were set at 365/480 nm for KYN and 344/398 nm for KYNA, respectively. The mobile phase comprised 250 mM zinc acetate, 50 mM sodium acetate, and 2% acetonitrile (pH 6.2), delivered at 0.5 mL/min through a reverse-phase C18 column (Nexcol C18, 5 µm, 50 mm × 3.0 mm; Shimadzu, Tokyo, Japan) under isocratic conditions. Retention times were approximately 3 min for KYN and 7 Min for KYNA.

Tryptophan was quantified using the same fluoresce detector, ser at excitation/emission wavelengths of 254/404 nm. The mobile phase consisted of 100 mM zinc acetate and 3% acetonitrile (pH 4.2). Separation was performed using ZORBAX Eclipse AAA column (3.5 µm, 4.6 × 150 mm; Agilent, Santa Clara, CA, USA) with flow rate of 1.0 mL/min. The retention time for Trp was approximately 5 min.

3-HK was detected using electrochemical detection. Supernatants were injected onto an Adsorbosphere Catecholamine C18 reverse-phase column (3 µm, 4.6 mm × 100 mm; Fisher Scientific, Hampton, NH, USA). The mobile phase consisted of 0.27 mM EDTA, 8.9 mM heptane sulfonic acid, 9% triethylamine, 0.59% phosphoric acid, and 3% acetonitrile, delivered at 0.6 mL/min. Detection was performed using an LC-4C electrochemical detector (BASi, West Lafayette, IN, USA), operated at an oxidation voltage of 0.5 V, a sensitivity range of 1.0 nA, and a filter setting of 0.10 Hz. The retention time for 3-HK was approximately 11 min.

### 2.6. Statistical Analysis

Data were presented as mean ± standard error of the mean (SEM). To evaluate pre- and post-intervention differences, the Wilcoxon signed-rank test for two paired samples was applied. As an exploratory study, the pairwise correlations among variables were assessed using Spearman’s correlation, where the *p*-value were not adjusted for multiple comparison. A *p*-value < 0.05 was considered statistically significant. The analyses were performed with GraphPad Prism 9.1.0 (GraphPad, San Diego, CA, USA).

## 3. Results

### 3.1. Participant Characteristics

A total of 24 women completed the study protocol and were included in the final analysis. The mean age of participants was 37.04 ± 6.38 years. Table 1 presents the baseline and post-intervention data for body composition, serum biochemistry, and liver function markers.

Following the 12-week WLP, participants demonstrated statistically significant improvements in body composition. Specifically, decreases were observed in total body weight, BMI, and body fat mass, both in absolute values (kg) and as a percentage. Despite these changes, participants continued to fall within the WHO criteria for obesity class I. Importantly, the percentage of fat-free mass increased significantly, indicating a positive shift in body composition, even though a modest but significant decrease in absolute muscle mass was also observed.

Metabolic parameters also showed favorable changes. Although reductions in fasting glucose, triglycerides, total cholesterol, LDL cholesterol, VLDL cholesterol, uric acid, bilirubin fractions (total, direct and indirect), AST, and ALT were noted, these changes did not reach statistical significance. However, a significant reduction in fasting insulin levels was observed, suggesting an improvement in insulin sensitivity. Additionally, serum levels of gamma-glutamyl transferase (GGT) and alkaline phosphatase (ALP) significantly decreased following the intervention. Nonetheless, these enzymes remained above standard clinical reference values, potentially indicating persistent metabolic dysfunction-associated liver disease. Interestingly, blood urea nitrogen (BUN) levels increased significantly, a change likely related to the 20% protein diet and enhanced protein catabolism induced by regular physical activity. Systemic inflammation appeared to improve, as evidenced by a significant reduction in serum C-reactive protein (CRP) levels. Conversely, cortisol levels increased significantly, although they remained within clinical acceptable limits, possibly reflecting an adaptative physiological response to exercise and caloric restriction.

### 3.2. Kynurenine Pathway Metabolites in the Serum Before and After the WLP

**Figure 1:**
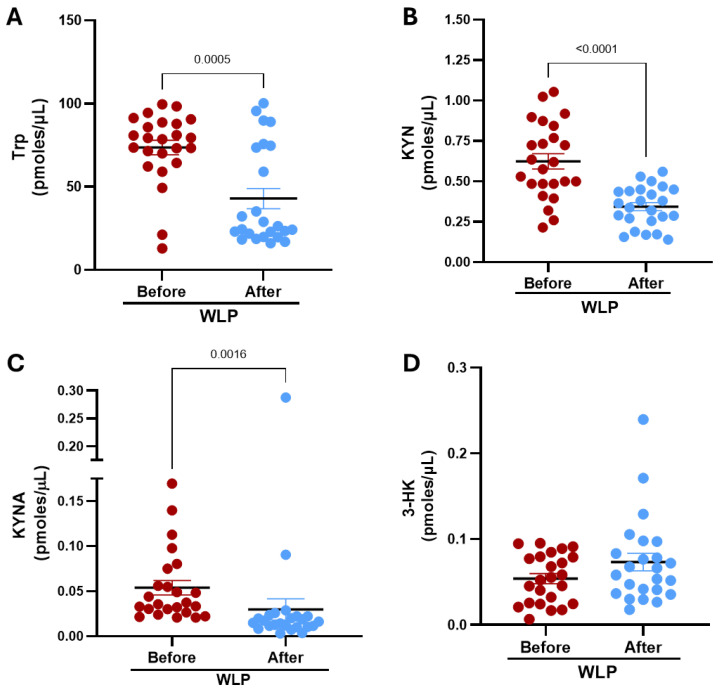
Effect of the WLP on serum KP metabolites in women with obesity: (**A**) Trp levels, (**B**) KYN levels, (**C**) KYNA levels and (**D**) 3-HK levels, measured before and after the 12-week intervention. Data are presented as mean ± SEM. *p*-values are based on the Wilcoxon signed rank test.

We next analyzed fasting serum concentrations of key KP metabolites at baseline and following three months of participation in the WLP. As shown in Figure 1, several metabolites exhibited statistically significant reductions after intervention. Specifically, Trp levels decreased by approximately 42%, from 73.59 ± 4.36 at baseline to 42.9 ± 6.06 pmol/µL post-intervention. A similar pattern was observed for KYN, which declined by approximately 45% (0.62 ± 0.05 vs. 0.34 ± 0.03 pmol/µL). KYNA also showed a significant reduction of about 45% decreasing from 0.05 ± 0.01 to 0.03 ± 0.01 pmol/µL. In contrast, 3-HK levels did not change significantly. However, a non-significant upward trend in 3-HK concentrations was noted at three months, which may indicate a potential shift within the KP metabolism.

**Figure 2:**
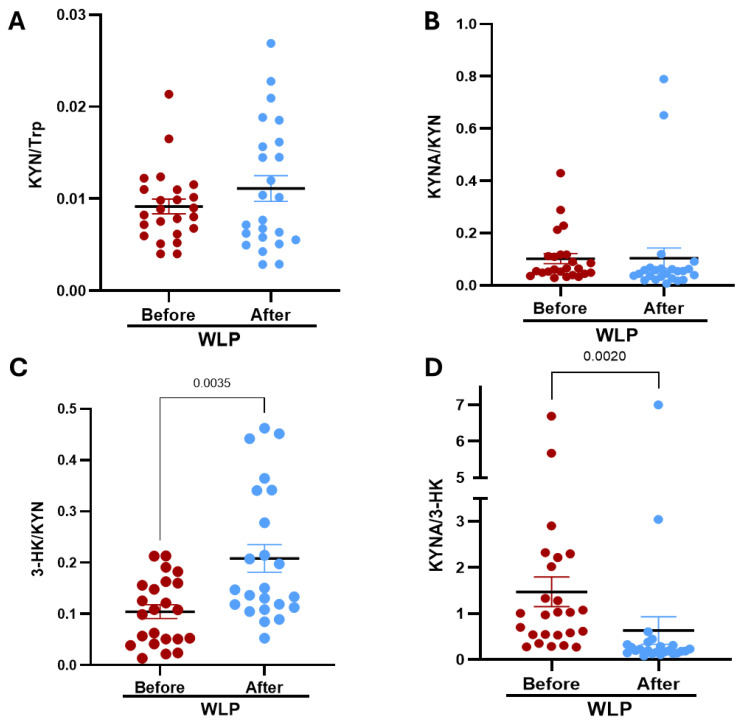
Effect of a 12-week WLP on serum KP metabolite ratios in women with obesity. (**A**) KYN/Trp ratio; (**B**) KYNA/Trp ratio; (**C**) 3-HK/Trp ratio; (**D**) KYNA/3-HK ratio. Data are presented as mean ± SEM. *p*-values are based on the Wilcoxon signed rank test.

To further explore changes in KP activity, we analyzed specific metabolites as ratios: KYN/Trp, a commonly used index of TDO or IDO activity; KYNA/KYN, indicative of KATs activity; 3-HK/KYN, reflecting kynurenine 3-monooxygenase (KMO) activity; and KYNA/3-HK, which serves as an indicator of the balance between arms of the KP and the relative proportions of these metabolites. No significant differences were observed in the KYN/Trp or KYNA/KYN ratios between baseline and post-intervention. In contrast, the 3-HK/KYN ratio was significantly increased three months after the intervention, indicating enhanced KMO activity. This shift was accompanied by a significant decrease in the KYNA/3-HK ratio (Figure 2).

### 3.3. Correlation Analysis of Kynurenine Pathway Metabolites with Clinical Parameters: Basal, Post-Intervention and as Relative Change

**Figure 3:**
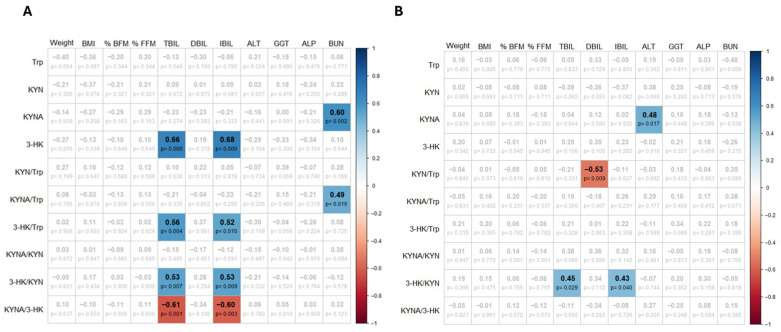
Heatmaps showing significant pairwise correlations between KP metabolites, body composition, and serum biochemistry (*p* < 0.05) before (**A**) and after (**B**) the WLP intervention. Color and +/− symbols indicate the direction and magnitude of the correlations. The *p*-values are not adjusted for multiple comparisons.

After observing significant changes in KP metabolites following the intervention, we explored potential associations between these metabolites and clinical parameters, including weight, BMI, fat mass percentage, fat free mass percentage, and liver function markers. We first performed correlation analyses to assess whether KP metabolites were associated with clinical and biochemical characteristics prior to the intervention. Interestingly, metabolites from the large arm (KMO arm) of the KP, specifically 3-HK and the ratios 3-HK/Trp and 3-HK/KYN, showed significant positive correlations with total bilirubin and indirect bilirubin, suggesting a potential link between KMO activity and hepatic metabolism. Conversely, the short arm (KATs arm) of the pathway, represented by KYNA and the KYNA/Trp ratio, was positively correlated with BUN levels, which may reflect the interaction between protein catabolism and Trp availability (Figure 3A).

Based on these findings, we further explored the associations after the intervention. Interestingly, we observed that KMO-related activity, as indicated by the ratio 3-HK/KYN, remained positively correlated with total and indirect bilirubin, reinforcing a persistent relationship between the KMO branch of the KP and hepatic bilirubin metabolism. In contrast, KYNA showed a positive correlation with ALT levels after the intervention, indicating a potential link between the KAT branch of the KP and hepatic enzyme activity under conditions of dietary and metabolic modulation (Figure 3B).

**Figure 4:**
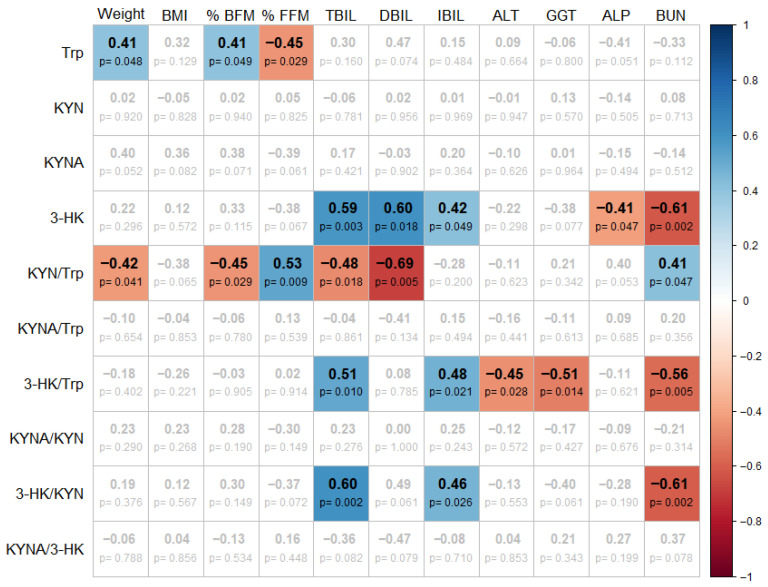
Heatmap showing significant pairwise correlations between the relative changes in KP metabolites, body composition, and serum biochemistry (*p* < 0.05). Color and +/− symbols indicate the direction and magnitude of the correlations. The *p*-value are not adjusted for multiple comparisons.

To account for individual variability in baseline body composition and metabolic profiles, we analyzed the relative change for each parameter evaluated (Figure 4). Using this approach, we found that the relative change in Trp was positively correlated with both weight and fat mass and negatively correlated with the change in fat-free mass. These findings suggest that greater reductions in Trp may be associated with more favorable changes in body composition, particularly reductions in adiposity.

For the KYN/Trp ratio, which reflects IDO/TDO enzymatic activity, we observed negative correlations with weight, fat mass percentage and bilirubin levels, indicating that an increment in this ratio may be associated with improvements in both metabolic status and liver function. Additionally, the KYN/Trp ratio was positively correlated with the change in fat-free mass and BUN. This may reflect an increased flux through the KP in response to heightened protein turnover and shifts in body composition.

Markers of KMO branch of the KP, specifically 3-HK and its ratios 3-HK/Trp and 3-HK/KYN showed positive correlation with bilirubin levels, while displaying negative correlation with BUN. Notably, 3-HK/Trp also exhibited negative correlations with hepatic enzymes ALT and GGT.

### 3.4. Correlation Analysis Between Relative Changes in Anxiety, Depression and KP Metabolites

**Figure 5:**
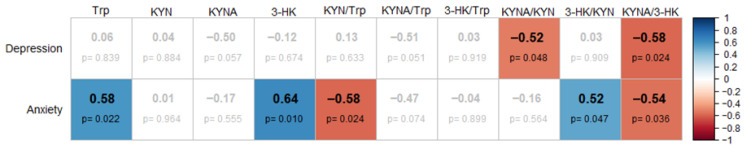
Heatmap showing significant pairwise correlations between the relative changes in KP metabolites and anxiety and depression (*p* < 0.05). Color and +/− symbols indicate the direction and magnitude of the correlations. The *p*-values are not adjusted for multiple comparisons.

Given that increased activity of KP enzymes may enhance the peripheral production of KYN, which can cross the blood–brain barrier (BBB) and be metabolized into KYNA in the central nervous system, it has been proposed that this mechanism could contribute to cognitive and behavioral alterations, including symptoms of anxiety and depression. In this study, a subset of 15 women with obesity from the naval cohort completed standardized psychological assessments for anxiety and depression both at baseline and after the 12-week intervention. The relative change in anxiety and depression scores was calculated for each individual, and correlation analyses were performed to assess potential associations with changes in serum KP metabolites. As illustrated in Figure 5, positive correlations were observed between anxiety levels and serum levels of Trp, 3-HK and the 3-HK/KYN ratio. Interestingly, the KYN/Trp and KYNA/3-HK ratios were negatively correlated with anxiety scores, indicating that a metabolic shift in the periphery favoring the KATs arm could prevent the enhancement of KYNA production in the brain and, consequently, reduce anxiety. Regarding depression, significant negative correlations were identified between depression scores and the KYNA/KYN and KYNA/3-HK ratios, implying that higher relative KYNA production may be associated with improvement in depressive symptoms.

## 4. Discussion

In this study, we investigated the metabolic adaptations in serum KP metabolites in a cohort of Mexican naval women with obesity who underwent a structured three-month weight loss program combining a hypocaloric diet and moderate-intensity exercise. This intervention led to significant changes in body composition and serum biochemical parameters, which occurred in parallel with modulations in KP metabolites profiles.

Our findings confirm that WLP induced statistically significant weight loss, characterized by reductions in BMI and total fat mass, along with an increase in fat-free mass percentage. However, participants remained within WHO-defined obesity class I, suggesting that while beneficial, the intervention resulted in partial rather than full metabolic normalization. These results are consistent with previous studies indicating that hypocaloric diets combined with physical activity are more effective for achieving negative energy balance and reducing body weight than exercise alone [27,28,29]. A modest loss in muscle mass was observed, as expected with calorie restriction, through exercise may have mitigate this effect [30,31].

In addition to anthropometric changes, the intervention led to modest but favorable shifts in metabolic and hepatic markers, including significant reductions in fasting insulin and CRP levels. Although changes in glucose, lipid profile, bilirubin, and liver enzymes such as AST, and ALT did not reach statistical significance, the trend toward improvement supports a systemic enhancement in metabolic function. The observed decline in fasting insulin is consistent with improved insulin sensitivity, particularly in skeletal muscle and liver, as previously documented in weight loss and exercise interventions [32]. Multiple mechanisms likely contributed to this outcome. During exercise, skeletal muscle increases glucose uptake independently of insulin by translocating GLUT4 transporters to the plasma membrane, enhancing peripheral glucose clearance [33]. Moreover, repeated bouts of exercise improve insulin signaling cascades, enhancing the responsiveness of insulin receptors and downstream intracellular targets [34]. Additionally, the reduction in adiposity, a major source of pro-inflammatory cytokines, likely contributed to the decrease in systemic inflammation, as reflected by lower CRP levels, further supporting improved insulin action [35]. Together, these mechanisms suggest that exercise enhances glucose regulation by increasing muscle glucose uptake, optimizing insulin signaling efficiency, and reducing inflammation associated with excess adiposity.

The WLP also resulted in a downward trend in hepatic enzymes (AST, ALT, GGT, and ALP) suggesting improved hepatic function, consistent with prior reports showing that caloric restriction and moderate exercise lead to decreased hepatic enzyme activity and reduced hepatic steatosis [36]. Despite these improvements, GGT and ALP levels remained elevated beyond clinical reference limits, suggesting persistent hepatic dysfunction, most likely attributable to non-alcoholic fatty liver disease (NAFLD) or residual inflammatory processes in extrahepatic tissues, such as biliary or adipose structures. These findings are aligned with evidence that moderate weight loss may not be sufficient to fully reverse NAFLD, particularly in the absence of prefund reductions in visceral fat or extended intervention periods [36]. GGT, an enzyme integral to glutathione metabolism, is particularly sensitive to oxidative stress and lipid peroxidation. Its sustained elevation may reflect ongoing redox imbalance, hepatic fat accumulation, or subclinical inflammation, despite overall metabolic improvements [37]. Likewise, elevated ALP may indicate residual cholestasis or hepatic remodeling processes, which often resolve more slowly than transaminase normalization [36]. Taken together, these findings indicate that caloric restriction and moderate-intensity exercise are effective in initiating hepatic recovery, but full normalization of liver function biomarkers, particularly GGT and ALP may require more intensive interventions, including greater fat loss, longer program duration, and potentially target strategies to restore redox homeostasis and bile acid metabolism.

A major and novel finding of this study is the coordinated shift in the KP metabolism observed in women with obesity following a 12-week structured WLP. This shift was marked by reductions in circulating levels of Trp, KYN, and KYNA, and a non-significant upward trend in 3-HK, collectively reflecting a reorientation of KP flux toward the KMO branch. These changes mirror previous reports describing similar reductions in Trp and KYN after caloric restriction, bariatric surgery, or structured exercise programs, where such shifts are attributed to reduced systemic inflammation, altered substrate availability, and increased peripheral uptake of these metabolites [38,39,40]. Trp reductions, in particular, is often interpreted as a result of IDO/TDO activation in response to residual metabolic stress, which aligns with the fact that participants in this study remained obese post-intervention [41,42]. Moreover, increased Trp catabolism may also be driven by enhanced amino acid use for gluconeogenesis, explaining the concurrent loss of fat-free mass and increase in BUN, a pattern previously reported in caloric restriction studies [43,44].

While absolute metabolite levels decreased, enzymatic activity indices provided critical mechanistic insights. The KYN/Trp ratio, a marker of IDO/TDO activity, showed a non-significant increase, possibly indicating sustained hepatic Trp degradation to maintain redox balance and NAD+ biosynthesis under persistent low-grade inflammation. This is further supported by persistently elevated GGT and ALP, markers of oxidative and hepatic stress [18,45,46]. Importantly, the 3-HK/KYN ratio increased significantly, denoting a metabolic shift toward the KMO branch, which is involved in NAD^+^ generation and redox adaptation [38]. The lack of change in KYNA/KYN ratio, a proxy for KATs activity, and the reduction in the KYNA/3-HK ratio both reinforce this preferential rerouting on KP flux. While increased 3-HK is sometimes associated with oxidative stress, in the context of weight loss and enhanced mitochondrial activity, this pattern may instead reflect improved metabolic flexibility and mitochondrial efficiency, consistent with previous findings of enhanced lipid oxidation and thermogenesis following similar interventions [14,15,47]. These metabolic rearrangements resemble those seen in obesity-associated inflammation, where KMO is upregulated in adipose and hepatic tissue, directing Trp catabolism away from KYNA and toward to the long arm intermediates [19,48]. Our data suggest that this flux redistribution may also occur as part of a beneficial adaptive response, rather than being strictly pathological.

To further explore the physiological relevance of KP modulation, we analyzed correlations between KP metabolites and hepatic markers, using relative changes to account for individual baseline variability. 3-HK and its ratios (3-HK/Trp and 3-HK/KYN) consistently correlated positively with total and indirect bilirubin. These findings suggest a functional link between KMO activity and hepatic bilirubin metabolism, which may reflect a shared role in redox homeostasis and detoxification, as bilirubin has potent antioxidant properties [49,50,51]. In addition, KYNA and the KYNA/Trp ratio showed positive correlations with BUN at baseline, but not with hepatic enzymes or bilirubin. This suggests that KAT-mediated metabolism may be more responsive to systemic protein catabolism and nitrogen disposal, rather than directly influenced by hepatic metabolic stress [30,49]. The post-intervention correlation between KYNA and ALT may indicate compensatory hepatic responses, as ALT reduction is a well-established marker of improvement in hepatic steatosis and hepatocellular stress [49]. These associations support the view that KMO- and KAT-branches of the KP are differentially regulated during metabolic adaptations, with KMO arm more tightly linked to hepatic redox processes, while KAT arm may be more influenced by protein turnover and extrahepatic factors.

Also, in support of the functional relevance of KP shifts, we observed that greater reductions in circulating Trp were associated with reductions in body weight and fat mass but inversely associated with fat-free mass. Similarly, the KYN/Trp ratio showed negative correlations with body weight, fat mass, and bilirubin, suggesting that increased KYN/Trp could serve as an integrative marker of metabolic recovery. Collectively, these findings suggest that moderate weight loss driven by lifestyle intervention partially restores KP homeostasis, promoting metabolic and hepatic shift toward improved redox function and substrate utilization. This underscores the emerging concept that KP operates as metabolic hub linking inflammation, amino acid catabolism, and hepatic function [49,52].

**Figure 6:**
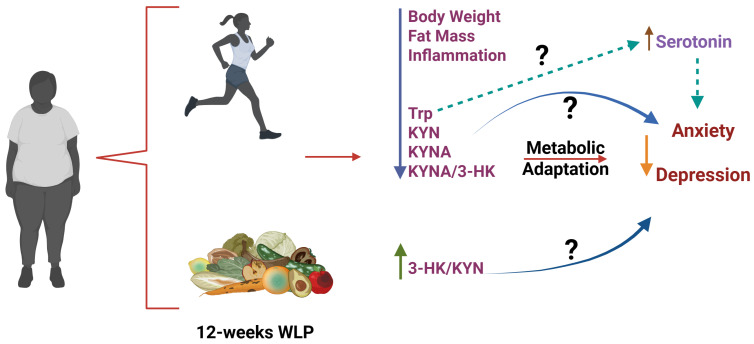
Proposed model illustrating the modulation of the kynurenine pathway in response to a structured weight loss program in obese Mexican naval women. The intervention, consisting of a hypocaloric diet and moderate-intensity exercise, was associated with reduced body weight, decreased inflammation and changes in tryptophan (Trp) metabolism. Changes in the KP suggest a shift toward the KMO axis, as indicated by increased 3-hydroxykynurenine (3-HK) and a reduced kynurenic acid/3-HK (KYNA/3-HK) ratio, which may relate to improvements in emotional well-being. Additionally, the observed reduction in circulating Trp might reflect increased uptake of this amino acid into the brain for serotonin synthesis, potentially contributing to reduced symptoms of depression and anxiety. These associations are exploratory and warrant further investigation.

In a subset of 15 participants, psychological assessments revealed significant associations between KP modulation and changes in anxiety and depression scores. Specifically, the KYNA/KYN and KYNA/3-HK ratios were negatively correlated with changes in depressive symptoms, while anxiety scores were positively correlated with changes in Trp, 3-HK and 3-HK/KYN, and negatively correlated with the KYN/Trp and KYNA/Trp ratios. These findings suggest that favoring the short arm of the KP, which converts KYN into peripherally acting KYNA, may help reduce emotional symptoms (Figure 6). In this sense, the reduction in KYNA levels observed after the WLP intervention and the improvement in depressive symptoms is consistent with a previous study of our group which demonstrated higher serum levels of KYNA in Mexican patients with post-stroke depression [53]. In contrast, greater KMO flux reflected by increased 3-HK, may be associated with elevated anxiety [54]. Exercise has been shown to upregulate skeletal muscle KAT expression, promoting peripheral conversion of KYN into KYNA and reducing its transport into the brain, where it may otherwise be converted into central KYNA, a metabolite associated with cognitive disruption and affective disorders [54]. Additionally, dietary modulation of KP activity has been associated with increased peripheral KYNA levels and lower depression scores [55]. Beyond its catabolism via the KP, Trp also serves as the sole precursor for serotonin synthesis, a neurotransmitter essential for regulating mood, anxiety, and cognitive function [56]. Exercise has been shown to modulate serotonergic signaling by increasing availability of free Trp and enhancing its transport across the blood–brain barrier, thereby promoting central serotonin production [57,58]. This mechanism may partially explain the improved mood and emotional well-being reported by participants, despite the observed reduction in circulating Trp levels.

This exploratory study has several limitations. First, the small sample size and the pre–post design without a control group limit statistical power and preclude causal inference. Second, the correlation analyses were based on *p*-values that were not adjusted for multiple comparisons, mainly due to the exploratory nature of this study and the small sample size. Third, the three-month duration may not be sufficient to observe long-term metabolic or psychological adaptations. Fourth, oxidative stress biomarkers were not measured, which limits our interpretation of KP shifts as compensatory or pathological. Fifth, serotonin levels were not measured, which limits our ability to assess the contribution of serotoninergic modulation to the observed emotional and behavioral improvements. Sixth, although dietary counseling was provided to ensure an adequate and stable intake of Trp, no direct quantification of dietary Trp was performed, which may influence circulating metabolite levels. Seventh, the menstrual cycle phase was not controlled for, which could introduce variability, although all participants were premenopausal women within a narrow age range. Eighth, the subsample used for psychological assessments was relatively small, limiting the generalizability of these specific findings and the power to detect subtle behavioral effects. Finally, the study cohort comprised only adult Mexican Naval women with obesity, which may restrict the generalization to other populations.

## 5. Conclusions

This exploratory study suggests that the KP may function as a dynamic metabolic hub responsive to lifestyle-induced weight loss. A 12-week intervention combining a hypocaloric diet and moderate-intensity exercise was associated with improvements in body composition and reductions in systemic inflammation, along with changes in Trp metabolism, which favored the KMO branch over KYNA synthesis. While causality cannot be established, the observed associations point to a potential role of the KP in coordinating protein catabolism and both liver and metabolic adaptation during weight loss. These preliminary findings add to the growing body of evidence that non-pharmacological modulation of KP activity may support both metabolic and physiological improvements in obesity management. Further studies with larger, controlled cohort and longer follow-up are needed to confirm and expand upon these results.

## Figures and Tables

**Table 1:** Body composition, serum biochemical parameters, and liver function markers before and after the WLP. Data are mean ± SEM. *p*-values are based on the Wilcoxon signed-rank test for paired samples.

Variable	Before	After	*p*-Value	Clinical Reference Limits
Body composition analysis				
Weight (Kg)	89.59 (2.13)	80.4 (2.01)	**<0.001**	
BMI (kg/m^2^)	35.04 (0.7)	31.39 (0.68)	**<0.001**	18.5–24.9 normal
				25–29.9 overweight
				30–34.9 obesity I
				35–39.9 obesity II
				40–49.9 obesity III
Body fat (%)	45.52 (0.85)	40.76 (1.03)	**<0.001**	Female < 35
Body fat mass (Kg)	41.13 (1.56)	32.67 (1.47)	**<0.001**	-
Free body fat mass %	54.48 (0.85)	59.24 (1.03)	**<0.001**	-
Free body fat mass (Kg)	47.54 (0.78)	46.96 (0.8)	**0.018**	-
Muscle mass (Kg)	24.68 (0.71)	24.24 (0.73)	**0.009**	-
Serum Biochemistry				
Glucose (mg/dL)	94.87 (2.99)	94.09 (2.09)	0.83	65–95
HDL-cholesterol (mg/dL)	44.38 (1.93)	43.67 (1.33)	0.974	40–60
LDL cholesterol (mg/dL)	127.54 (8.72)	123.49 (5.9)	0.855	<129
VLDL cholesterol (mg/dL)	23.92 (1.54)	21.99 (1.68)	0.214	2–30
Total cholesterol (mg/dL)	195.96 (10.01)	188.71 (7.45)	0.372	<200
TG (mg/dL)	122.17 (8.27)	109.83 (8.41)	0.121	<150
C-reactive protein (mg/dL)	0.68 (0.15)	0.37 (0.1)	**<0.001**	0–0.8
Uric acid (mg/dL)	5.44 (0.2)	5.37 (0.19)	0.647	2.5–5.6
Insulin (Uu/mL)	11.18 (0.98)	8.34 (1.16)	**0.02**	2–20
BUN (mg/dL)	10.77 (0.59)	13.67 (0.89)	**<0.001**	7–20
Cortisol (µg/dL)	6.85 (0.73)	9.08 (0.61)	**0.006**	8.7–22.4
Liver panel				
Total bilirubin (mg/dL)	0.76 (0.05)	0.73 (0.05)	0.569	0.2–1
Direct bilirubin (mg/dL)	0.07 (0.01)	0.09 (0.01)	0.239	0–0.2
Indirect bilirubin (mg/dL)	0.7 (0.04)	0.65 (0.05)	0.362	0–0.85
AST (UI/L)	24.96 (2.22)	21.04 (0.97)	0.116	10–42
ALT (UI/L)	33.14 (5.33)	22.18 (1.35)	0.06	10–40
GGT (UI/L)	23.43 (2.38)	14.83 (1.1)	**<0.001**	8.37
ALP (UI/L)	61.63 (3.02)	54.49 (2.11)	**0.005**	32.92

Body mass index, BMI; Blood Urea Nitrogen, BUN, Triglycerides, TG; Aspartate aminotransferase, AST; Alanine aminotransferase, ALT; Gamma-glutamyl transferase, GGT; Alkaline phosphatase, ALP.

## Data Availability

The raw data supporting the conclusions of this article will be made available by the authors on request.

## Abbreviations

The following abbreviations are used in this manuscript:

3-HK	3-hydroxykynurenine
AA	Anthranilic acid
AhR	Aryl hydrocarbon receptor
ALP	Alkaline phosphatase
ALT	Alanine aminotransferase,
AST	Aspartate aminotransferase
BBB	Blood–brain barrier
BMI	Body mass index
cLGI	Chronic low-grade inflammation
CRP	Serum C-reactive protein
GGT	Gamma-glutamyl transferase
GPR35	G-protein-coupled receptor 35
HCl4	Perchloric acid
HPLC	High-performance liquid chromatography
IDO1	Nicotinamide adenine indoleamine 2,3-dioxygenase 1
IDO2	Nicotinamide adenine indoleamine 2,3-dioxygenase 2
IFN-γ	Interferon-gamma
IL-6	Interleukin-6
ISAK	International Society for the Advancement of Kinanthropometry
KATs	Kynurenine aminotransferases
KMO	Kynurenine 3-monooxygenase
KP	Kynurenine pathway
KYN	Kynurenine
KYNA	Kynurenic acid
LPS	Lipopolysaccharides
METs	Metabolic Equivalent of Task units
NAD^+^	Nicotinamide adenine dinucleotide
NAFLD	Non-alcoholic fatty liver disease
PA	Picolinic acid
QUIN	Quinolinic acid
SECTEI	Secretaría de Educación, Ciencia, Tecnología e Innovación
SEM	Standard error of the mean
STAT3	Signal transducer and activator of transcription 3
TDO	Tryptophan 2,3-dioxygenase
TNF-α	Tumor necrosis factor alpha
Trp	Tryptophan
WAT	White adipose tissue
WHO	World Health Organization
WLP	Weight loss program
XA	Xanthurenic acid
